# Faculty retention in regional medical schools in Iran: a qualitative content analysis

**DOI:** 10.1186/s12909-020-02473-y

**Published:** 2021-01-06

**Authors:** Maria Shaterjalali, Yousef Gholampoor, Ali Khani Jeihooni, Yaser Mansoori, Reza Homayounfar, Elham Ehrampoush, Shahnaz Karimi

**Affiliations:** 1 Medical Science School, Islamic Azad University of Tonkabon Branc, Tonekabon, Iran; 2 Department of Internal Medicine, Fasa University of Medical Sciences, Fasa, Iran; 3 Department of Public Health, Fasa University of Medical Sciences, Fasa, Iran; 4 Department of Medical Genetics, Fasa University of Medical Sciences, Fasa, Iran; 5 Department of Nutrition, Fasa University of Medical Sciences, Fasa, Iran; 6 Medical Education Research Center, Fasa University of Medical Sciences, Fasa, Iran

**Keywords:** Attrition, Faculty, Retention

## Abstract

**Background and purpose:**

Recruitment and retention of competent faculty members are important in maintaining and improving the quality of education and research performance of universities. The aim of the present study was to find out the faculty members’ views, experiences, and attitudes to identify the reasons for faculty attrition and retention in regional medical schools in Iran.

**Methods:**

In this qualitative study, we used a content analysis method. The participants included 12 faculty members who had been transferred to type I universities, four faculty members who had applied for transfer, four with more than 10 years of experience and working in the type 3 universities with no intention to be transferred. Data were collected using semi-structured interviews, which were conducted either face-to-face or via phone calls. The interview was developed for this study (Supplementary file). To measure the trustworthiness of the data, we evaluated four components of credibility, transferability, dependability, and conformability, as proposed by Lincoln and Guba.

**Results:**

The findings were classified into three categories and 14 subcategories. The first category was “retention facilitators” including four subcategories of facilitated communication, proximity to major universities, gaining experience, and support by authorities. The second category was “retention threats” including six subcategories of social infrastructure, individual dimension, occupation dimension, economic dimension, sense of respect, and executive management. The third category was “retention strategies” which included four subcategories of recruitment and promotion processes, inter-university collaboration with type I universities, facilitation of the scientific growth, and fulfilment of the safety needs.

**Conclusion:**

Several factors play a role in the faculty members’ retention in regional medical schools in Iran. Authorities can create a more positive environment by devising a suitable reward system, supporting academic activities, and increasing the level of faculty autonomy practically to develop a sense of belonging among them and reduce the intention to be transferred among their human resources.

**Supplementary Information:**

The online version contains supplementary material available at 10.1186/s12909-020-02473-y.

## Background

Faculty members, as the cornerstone of education in medical sciences [1], are considered important assets for any higher education institution [2]. Ranking of universities in both national and international arenas also depends on the competence of the faculty member [3]. Recruitment and retention of talented faculty members are important in maintaining and improving the quality of education and research performance of the universities [4]. One of the challenges with which regional medical schools are faced is recruitment of faculty members, which can impose a significant burden on both individuals and institutions due to its complexity and difficulty [1]. An even more important challenge is the transfer or attrition of faculty members. According to a study conducted in the United States, five out of ten faculty members were transferred during 10 years [5]. In this regard, Fang and Bednash (2014) reported that 11.8% of full-time faculty members in 2010 left their full-time jobs by 2011 [6].

Concerns about the faculty retention is usually rooted in the fact that the transfer of the faculty members imposes heavy expenses on the universities due to the loss of recruitment investment, recruitment of new faculty members, and the long processes of recruitment. Moreover, the negative impact of faculty substitution and early transfer can interrupt the process of education and counseling, with major consequences for the academic system [1, 7]. On the other hand, the criteria for faculty retention are often vague, and there is no definite mechanism for retaining faculty members in the universities [2].

Various studies in other countries have been conducted in higher education institutions to identify the factors affecting the faculty retention. In this regard, Soomro and Ahmad (2013) suggested some strategies in the field of education, research, and community services to retain the faculty [2]. Breslow, on the other hand, showed that some faculty members were transferred due to personal reasons, lack of supportive working environments, and better offers elsewhere [8]. Lee (1982) found that faculty attrition for personal reasons, job dissatisfaction, and low salary was slightly higher at the state universities, compared to social and technical sciences schools [9].

Structured programs seem to be effective in faculty members’ promotion, and enhancement of the quality of students’ learning and progress can lead to faculty retention. Ries et al. (2012), in a study at the National Center of Leadership in Academic Medicine (NCLAM) of the University of California, found that programs, including professional development workshops, strategic planning, individualized academic performance counseling, mentoring with a senior faculty member for professional development, and creating a network with other faculty members for promoting assistant professors in health sciences had significant effects on the retention of newly employed assistant professors [1]. Locke also found that faculty members in different areas had different attitudinal and behavioral patterns shaped by their distinct epistemology, organizational commitment, and social relationships [10] and could be highly effective in faculty retention. Therefore, universities need to design and implement programs for the complex processes of faculty recruitment to maintain their human resources and reduce the negative consequences of faculty relocation.

There are 65 universities of medical sciences with 8000 faculty members and 200,000 students in the Ministry of Health and Medical Education in Iran at present. Faculty members are recruited in a competitive environment through recruitment recall after approval of the scientific and general competencies of candidates by authorities. Faculty members are recruited full-time geographically or full time non- geographically and in different recruiting forms, including formal, committed to service, soldier, and contractual [11].

Iranian university of medical sciences are evaluated and classificated into three types (1, 2, and 3) by the Department of Research and Technology of the Ministry of Health with determined criteria. Based on this categorization, top-ranking universities are type I, developing universities are type II, and newly established universities are type III universities. Type I universities include those located in major cities. Also, some type II or III universities are located in less developed or deprived cities. Iran Medical universities in face numerous challenges for faculty retention in deprives cities. Shortage of qualified faculty members, lack of consistency between number of faculty members and student, their lack of interest faculty members to continue working in deprived regions or less developed cities and Willingness to transfer to Type I universities are some of the challenges of the Ministry of Health in Iran. The highest rate of faculty transfer has been found among faculty members in type 3 universities, so employing and training new faculties is costly. This despite the fact that role of faculty members in increasing the university ranking is important due to their educational and research activities. Therefore, it is necessary to identify effective factors on faculty retention in medical schools. Additionally, an understanding of faculty members’ experiences on faculty retention and attrition is important; because it will help organizations identify the factors that threaten faculty retention and then quality of education [1].. By determining and understanding the root causes of faculty retention, a policy improvement can be created to increase faculty retention in deprived cities. Most studies on faculty retention have focused on specific academic groups used a quantitative method or have been done in other countries. Despite international and national interests in faculty retention, few have focused on medical schools and employed a qualitative approach. No qualitative study on regional medical schools to faculty retention has been done in our country. Because of our county’s socioeconomic cultural differences with other countries, the current study aimed to exploring faculty members’ views, experiences and attitudes on the faculty retention.

## Methods

### Study population and design

The main purpose of qualitative studies is to describe or discover a phenomenon, problem, or subject and examine a wide range of questions related to experiences, knowledge, attitudes, emotions, and perceptions of the individuals [12]. We conducted a qualitative content analysis study with the aim of determining the lived experiences of the subjects. Generally, content analysis is an approach which provides new knowledge and insights into a particular phenomenon. It presents valid inferences from data and is suitable for examining the experiences and views of people towards the issue of interest [13].

Purposive sampling method was done among the faculty members of medical schools of Iran from August 2018 to April 2019 with the maximum variation in terms of age, gender, academic rank, and work experience. Eligible people for the research were those who met the criteria and qualifications (At least 5 years of service in medical universities in deprived cities as faculty member) and were able to provide rich and complete information to the researcher.

The participants were carefully selected to obtain the richest information and reach data saturation. The subjects included 20 transferred faculty members, members who had applied for transfer, and those with more than 10 years of experience working in regional universities without any application for transfer although their families lived in large cities. We aimed to identify their experiences, tendencies, and views about the reasons for faculty retention and attrition.

For data collection, the participants were first identified and contacted for participation in the study. Before each interview, we explained the study objectives to the participants, and through agreement and obtaining their consent to participate, determined the time and place for the interview. Out of 20 interviews, 16 were conducted face-to-face, and four via phone calls. At the beginning of the interviews, we asked for the participants’ informed consent to record the interviews, and they were assured that their personal information would remain confidential.

The sampling process continued until theoretical saturation [12]. The interviews lasted for 45–60 min, starting with the following general question: “What do you think is the reason for faculty attrition in medical schools?” The interviews continued with questions, such as “What are the factors affecting faculty retention in medical schools?” and “What strategies do you suggest for encouraging the faculty members to remain at regional universities?”, and ended with follow-up questions. To analyze the data, we performed a qualitative content analysis with the inductive reasoning approach. To this end, the data were read repeatedly, and then semantic units were extracted. After summarizing the units, condensed semantic units were obtained, which were reduced to a limited number of words. These semantic units were semantically equivalent to the original semantic units.

Next, semantic units with similarities were converted into codes by labeling. At this stage, we used peer-checking to ensure the trustworthiness of data interpretations. The number of primary codes was 123, which was reduced to 90 after merging similar codes and removing identical ones. In the next stage, after comparing the codes, they were divided into subcategories. The subcategories were constantly compared and reviewed, so that we could merge or separate some of them based on common or different characteristics and form a new subcategory. In the final stage, subcategories, which seemed to have common characteristics, were merged in the main categories. The categories created were reviewed and discussed by five researchers (SHK, MSJ, YGH, AKH, and YM).

To measure the trustworthiness of data, we evaluated the four components of credibility, transferability, dependability, and confirmability, as proposed by Lincoln and Guba [14]. Credibility was evaluated through member checking and prolonged engagement with the data. The transcripts of interviews and extracted codes were presented to some of the participants (*n*=8), and they were asked to comment on the researchers’ perceptions of their statements and correct any incongruencies. Transferability was established through maximum variation sampling, which provided thick descriptions and straightforward examples of data. Dependability was also established as the participants provided similar answers to the same questions. Finally, for confirmability, we tried to avoid any bias towards the data as the findings were shaped by the respondents [14, 15].

This study was approved by the research ethics committee of Fasa University of Medical Sciences (Number of ethics code: IR.FUMS.REC.1398.044).

## Results

The study population consisted of 20 faculty members, including 12 males and 8 females; three of them were executive manager (dean of faculty, Vice Chancellor in Education, Vice Chancellor in Research). Also, 9 participants were from two schools, and 11 were from three universities. Interpretation of data revealed many factors concerning faculty retention in medical universities. The findings were divided into three categories and 14 subcategories. The results are presented in Table 1. Review of the participants’ views revealed some differences, which could be attributed to different conditions in departments or faculties of medical schools.

**Table 1:** Categories of faculty retention

Categories	Subcategories
Retention facilitators	Facilitated communication
	Proximity to type I universities
	Gaining experience
	Authorities’ support
Retention threats	Social infrastructure
	Individual dimension
	Occupational dimension (education and research)
	Economic dimension
	Sense of respect
	Executive management dimension
Retention strategies	Recruitment and promotion processes
	Inter-university collaboration
	Facilitating scientific growth
	Fulfilling welfare needs

The first category was “retention facilitators”, including four subcategories of facilitated communication, proximity to larger (type I) universities (the definition of university ranking is given at the end of the section), experience gained, and support by authorities. Summary of the participants’ views about these subcategories indicated facilitation of interpersonal relationships, work activities in a friendly atmosphere, increased cooperation, intimate communication, greater opportunities for junior faculty members to teach and get promotion, higher salaries, more efforts by authorities to solve the problems, and existence of administrative bureaucracy.

In subcategories of facilitated communication and gaining experience, the participants stated:

“*In type III universities, there is a more intimate atmosphere, and the activities are done more easily. The lower workload and convenient interactions with the university staff are the reasons for my retention.”* (Participant 4).

“*The education system was suitable for me. The living standards were excellent because we lived in the university campus; we could both live and work in the same place.”* (Participant 1).

The second category was “retention threats” including six subcategories of social infrastructure, individual dimension, occupational dimension, economic dimension, sense of respect, and executive management. The main threats for the faculty retention were social infrastructure and individual dimensions. The social infrastructure refers to under development or lack of adequate facilities, such as advanced training centers, recreational centers, and sports clubs in towns. Therefore, some faculty members needed to move away from their families and spend their weekends commuting from the family hometown to the workplace, which increases their tendency to transfer to other universities.

Summary of the participants’ views about these subcategories revealed the following results: lack of living and medical facilities in small cities; cultural differences among non-native faculty members; willingness of the faculty members to live with their families; lack of facilities in the faculty housing provided by universities; authorities’ negligence of the faculty members’ academic activities; dissatisfaction with increased administrative workload; poor performance of school officials in retention and recruitment of faculty members; inadequate facilities for the children of faculty members in small cities; greater opportunities for academic activities in type I universities; and better financial status of type I universities. As to the subcategories of social infrastructure, individual dimension, sense of respect, and executive management, some of the participants remarked:

“*What facilities have they provided for me to stay in the university? I regret having stayed there. Why shouldn’t I go to another university for a couple of years?”* (Participant 12).

“*The main reason I moved was my family.”* (Participant 10).

“*Some new technologies are available at type I universities.”* (Participant 6).

“*There is so much work to do and I do not even have the time to do my own work (education and research); for example, for retraining, I need to make sure that the workshop has been held.”* (Participant 16).

The third category was “retention strategies” including four subcategories of recruitment and promotion processes, inter-university collaboration with type I universities, facilitation of scientific growth, and provision of welfare issues. Summary of the participants’ views about the processes of faculty recruitment and promotion indicated the need for recruiting local faculty members (living in remote areas), planning for faculty recruitment during PhD or other specialty courses based on their resume, establishing longer periods of commitment, having commitment to an extended period of retention, facilitating the process of faculty recruitment, and shortening the time to promotion.

In addition, summary of the participants’ views in this area showed the following findings: need for communication with the faculty members of type I universities; encouraging research teams with the aim of using the facilities of type I universities; providing opportunities for faculty members to experience working in type I universities; offering doctoral and postdoctoral fellowships; macro-planning for introducing further non-financial incentives, such as educational workshops and sabbatical leaves; facilitating the promotion of the academic staff; improving communication with industry; providing opportunities for education and research development; allowing the clinical faculty members to perform their own academic activities; providing financial and non-financial incentives; increasing the salary of the faculty members; improving their accommodation; providing job security; and selecting committed school managers who would motivate the faculty members. In subcategories of facilitating the scientific growth, inter-university collaboration with type I universities and fulfilling welfare needs, some participants stated:

“*Residents of other universities should be identified, their resume and CV should be reviewed, and they should be employed in the university.”* (Participant 3).

*“They can be more flexible. A university which offers a Master’s or PhD course has professors with eight to ten published articles. However, it is important for them to write proposals and collect data in type III universities. I think promotions should be facilitated in underprivileged regions.”* (Participant 4).

“*It is possible to sign contracts with type I universities and use their facilities.”* (Participant 17).

“*Since faculty members are allowed to establish knowledge enterprises, universities should support them.”* (Participant 2).

“*A manager should first be able to manage himself/herself and be honest. Most managers are willing to be transferred to type I universities, so that they can have the opportunity for promotion. Managers should accept that their school is excellent and should be satisfied with it.”* (Participant 15).

## Discussion

The present study was carried out to explore faculty retention of regional medical schools in Iran. Based on the experiences of the participants in the present study, the factors affecting the retention of the faculty members include three main categories: retention facilitators, retention threats and retention strategies. Our findings as to the faculty retention in regional universities revealed more extensive threatening factors of faculty retention than facilitating factors. Another finding was the need to pay attention to the use of incentives for faculty retention. In this section, the categories that were most emphasized by the participants are discussed.

### Retention facilitators

In the present study, one of the main categories was retention facilitators, which included sub-categories such as facilitated communication, support by authorities, proximity to type I universities, and experience gained. In the area of support of authorities, Hendrickson et al. (2013) also points out that the satisfaction of faculty members was related to their role in fulfilling the goals of universities [16]. It seems that the measures taken by university authorities to establish favorable human relationships and focus on their institutionalization in the university atmosphere can pave the way for faculty retention, which requires the special attention of the management. In this regard, Dittmer (2017) showed that the university atmosphere and satisfaction of working with colleagues could affect the faculty retention [17].

Our results are consistent with the findings reported by Mirkamali et al. (2015) on providing a safe environment to facilitate the teamwork for employee retention [18]. Reduction of administrative bureaucracy, authorities’ efforts to solve the faculty members’ problems, and improvement of their quality of life were among the factors influencing the faculty members’ satisfaction and happiness. Overall, the welfare and educational facilities in deprived cities, supportive atmosphere of small universities, and positive attitude of the university management can create a safe environment and a sense of security, which encourage the retention of the faculty members. Most participants believed that educational facilities and welfare could provide a supportive environment and develop a sense of security, which eliminates their concerns about the future.

A study by Johnson showed that improving the faculty members’ quality of life and providing a high level of job satisfaction could improve the faculty members’ retention and promotion [19]. Therefore, it is crucial to pay special attention to the needs and requests of faculty members and solve their problems. Another retention facilitator is holding professional development programs and providing opportunities for the promotion of the faculty. It seems that establishment of individual development programs in line with the faculty’s professional background can contribute to the retention of human resources in universities. Also, the use of educational programs of type I universities can improve individual development and faculty retention. A study by Ries et al. indicated that faculty retention was dependent on job satisfaction and academic progress [1].

### Retention threats

The findings of the present study showed that another major category suggested by the faculty members was the “Retention threats”. Regarding the retention threats, sub-categories such as lack of necessary social infrastructures, besides individual, occupational, and economic aspects of the workplace, was among the factors affecting the transfer or substitution of faculty members. Lack of decent living facilities in cities eliminates the possibility of staying with family and encourages the faculty members’ transfer. The sense of belonging to a new environment is the basic need of every individual. Lack of a sense of belonging to the new environment is exacerbated by the absence of family and increases the faculty members’ tendency to transfer. On the other hand, factors, such as work climate, favorable urban environment, living facilities, accessibility of recreational centers, and possibility of private activities enable the faculty members to stay with their families, resulting in faculty retention.

In Iran, faculty members of basic and clinical sciences courses are considered full-time employees and are not allowed to work in other organizations unless they obtain permission from the university and the Ministry of Health. This includes private work activities outside the university (e.g. working in a personal office, pharmacy, laboratory, diagnostic center, educational center, charity organization, or private hospital). However, some faculty members do like the opportunity to have such private activities. We believe that by increasing the salary and benefits of faculty members and providing job security, we can reduce their need to pursue private activities and facilitate faculty retention in small universities.

Since changes in the urban infrastructure do not solely depend on universities, it is crucial that university administrators pay special attention to the faculty members’ cultural differences and desires while designing faculty recruitment policies and planning for recruiting local faculty members. Cultural convergence is one of the important issues that requires the authorities’ attention when recruiting faculty members. Cultural difference in this context means that faculty members who are recruited in type II or III universities in towns are faced with a difference between their own lifestyle and the new community. These individuals are concerned about cultural rejection and the future of their families. Therefore, they are often forced to live apart from their families and commute weekly from their place of residence to workplace, which can ultimately lead to faculty attrition.

In recruitment programs, it is advisable to consider the individual characteristics and desires of the applicants in all domains, including lifestyle, interests, and concerns. Furthermore, provision of good social welfare services at universities in remote cities is one of the issues that should be considered by university planners. For facilitating the economic growth of the university faculties (departments), it is important for the universities to be involved in the industry and reduce the restrictions on personal activities in basic sciences (University—Industry Relations). Lord and Farrington (2006) also show that employees need to be ensured that there is a safe and secure environment for their families in order to remain in a job [20]. Moreover, Lindfelt et al. (2018) found that the balance between work and life was effective in reducing the faculty members’ intention to change their job [21]. Lack of a sense of respect by officials was another issue threatening the faculty retention. In this regard, the results of a study by Horwitz et al. in Singapore are consistent with the present findings [22].

### Retention strategies

Another category emerging in the present study was retention strategies. Given the importance of faculty recruitment at schools, the use of appropriate methods and strategies for faculty retention is crucial. To improve faculty retention at schools, authorities must provide faculty members with opportunities for professional growth through promoting research activities and inter-university collaboration with type I universities. In type I universities, postgraduate students are usually residents, and professors can facilitate their own professional development through interactions with these students. However, conditions are not always as favourable in type II or III universities in underprivileged areas. The majority of the participants believed that if they could participate in academic and research programs of type I universities at least two days a week, their professional development would be facilitated.

Murugappan and Durga (2015) suggest that career and research opportunities strongly affect the faculty members’ retention [23]. Recruiting local faculty members and facilitating their promotion can be also one of the ways to retain faculty members. Our findings are consistent with the results reported by Nderitu (2014) regarding the impact of transparent policies on the recruitment, promotion, and retention of faculty members [24]. Also, the results of this study showed that longer commitments could affect the faculty retention. In this regard, Lavania et al. (2011) suggested flexible policies and programs for faculty retention [25]. Besides, it is crucial to pay special attention to the individual, economic, and social needs of faculty members.

This study had some limitations. Shortage of time of the research participants and the research environment in three universities were two limitations in the collection of data. We used telephone interview to reduce these limitations. Another limitation was the higher number of basic sciences faculty members than clinical sciences ones. Since Iran is a vast country with different cultural characteristics, further research is needed to explore the factors affecting the faculty retention.

## Conclusion

According to the present study findings, medical schools in deprived cities in Iran faced with many challenges in retention of faculty members. The role of authorities’ support in facilitating the faculty retention was significant. Authorities can establish an appropriate system for recruitment and promotion of faculty members, support their academic activities, build a proper collaborative network with type I universities, and design individual development plans, so that more faculty members can attend educational programs at type I universities. They should also create a more positive environment to foster a sense of belonging among human resources and reduce their intention to transfer. Overall, the solutions presented in this study can affect the further retention of faculty members. The comparison of the above findings with those of other studies suggests that the factors mentioned in this study are very important and could be taken seriously to improvement faculty retention policies in Iran and developing country like Iran that are faced with faculty attrition challenges.

## Supplementary Information

**Additional file 1.**

## Data Availability

Data that support the findings of this study are not publicly available but can be obtained from the authors on reasonable request. All data are available from the corresponding author on reasonable request.
