# Supplementary material for: Faculty retention in regional medical schools in Iran: a qualitative content analysis
Source: BMC Med Educ. 2021 Jan 6;21:24. doi: 10.1186/s12909-020-02473-y (PMC7788721; doi:10.1186/s12909-020-02473-y)
Supplement: Supplementary file 1 — Additional file 1. [file 12909_2020_2473_MOESM1_ESM.pdf]

Semi-structured interview guide

Participant No. Date:

The main questions addressed to faculty members who have been working at their place of employment in medical universities located in underprivileged areas for more than ten years.

1. You have been introduced to us by one of our colleagues to work as a faculty member at this university. Can you tell me about how you chose this university as your workplace?
2. What conditions or factors have played a role in your stay at this university?
3. What tactics do you suggest for attracting and retaining faculty members in underprivileged areas?

Main questions in interviews with faculty members applying to transfer to other universities:

1. You have been introduced by one of our colleagues as a faculty member applying to transfer to another university. We may go back to the time you were employed at this university. Talk to me then. How did you choose this university?
2. What conditions or factors made you decide to request for transfer?
3. What tactics do you suggest for attracting and retaining faculty members in underprivileged areas?

Main questions in interviews with faculty members transferred to other universities:

1. You have been introduced by one of our colleagues as a faculty member who has previously been at a university located in an underprivileged area. Go back to the time you were employed at the previous university and talk to me about it. How did you choose that university?
2. What factors made you decide to request for transfer?
3. What conditions or factors can contribute to the stay of faculty members in underprivileged areas?
4. What tactics do you suggest for attracting and retaining faculty members in underprivileged areas?
